# Visualization of Cellular Membranes in 2D and 3D Conditions Using a New Fluorescent Dithienothiophene *S,S*-Dioxide Derivative

**DOI:** 10.3390/ijms24119620

**Published:** 2023-06-01

**Authors:** Aneta Rzewnicka, Jerzy Krysiak, Róża Pawłowska, Remigiusz Żurawiński

**Affiliations:** 1 Division of Organic Chemistry, Centre of Molecular and Macromolecular Studies, Polish Academy of Sciences, Sienkiewicza 112, 90-363 Lodz, Poland; aneta.rzewnicka@cbmm.lodz.pl (A.R.); jerzy.krysiak@cbmm.lodz.pl (J.K.); 2 Division of Bioorganic Chemistry, Centre of Molecular and Macromolecular Studies, Polish Academy of Sciences, Sienkiewicza 112, 90-363 Lodz, Poland

**Keywords:** bioimaging, fluorescent dye, membrane staining, dithienothiophene

## Abstract

Cellular membranes play a key role in cell communication with the extracellular environment and neighboring cells. Any changes, including their composition, packing, physicochemical properties and formation of membrane protrusions may affect cells feature. Despite its great importance, tracking membrane changes in living cells is still a challenge. For investigation of processes related to tissue regeneration and cancer metastasis, such as the induction of epithelial-mesenchymal transition, increased cell motility, and blebbing, the possibility to conduct prolonged observation of membrane changes is beneficial, albeit difficult. A particular challenge is conducting this type of research under detachment conditions. In the current manuscript, a new dithienothiophene *S*,*S*-dioxide (DTTDO) derivative is presented as an effective dye for staining the membranes of living cells. The synthetic procedures, physicochemical properties, and biological activity of the new compound are presented herein. In addition to the labeling of the membranes in a monolayer culture, its usefulness for visualization of membranes under detachment conditions is also demonstrated. Obtained data have proven that a new DTTDO derivative may be used to stain membranes in various types of experimental procedures, from traditional 2D cell cultures to unanchored conditions. Moreover, due to the specific optical properties, the background signal is reduced and, thus, observation may be performed without washing.

## 1. Introduction

Cellular membranes are one of the most important cell components, involved in numerous processes required for the proper functioning of cells such as endocytosis, migration, cell division, intracellular communication, and the reception of extracellular signals [1,2]. Development, tissue regeneration, as well as cancer progression and metastasis, are related to membrane rearrangements and changes in its motility and physicochemical properties. Any alterations in membrane composition, structure, morphology, viscosity, and even shape, may be connected with numerous pathological states [3]. The key examples for visualizing the importance of membrane-related changes in cell properties are cancer cells. It was frequently demonstrated that cancerogenesis and, subsequently, cancer progression and metastasis are related to various alterations in membrane structure, composition, and characteristics [4,5]. The quantity and type of transmembrane proteins, including surface receptors, enzymes, and other elements, affect extracellular signaling and may induce critical changes in cell properties. The presence of receptors for extracellular nucleotides [6,7,8] and growth factors [9,10,11] may induce changes related to acquiring an invasive character of cancer cells. This type of cell communication with the surrounding area may provide induction of epithelial-mesenchymal transition, anoikis resistance, and enhancement of cell migration [12,13].

Numerous data have shown that not only constitution, but also membrane fluidity [14] and shape [15], are important factors which may play a key role in various physiological and pathological processes. The formation of membrane protrusions is an important element of cell functioning. These kinds of changes were originally discovered to be associated with cell death [16]; however, further investigations have also shown their importance in other processes related to cell movement [17,18,19,20,21] during chemotaxis [22] and invasion [15,23,24], as well as those related to anoikis resistance [25,26]. Although membrane blebbing was originally discovered as a process related to apoptosis, the newest data indicate that it may be connected with the escape of other types of cell death induced by detachment conditions named anoikis [25,27]. Thus, both amoeboid cell movement and anoikis resistance may depend on the blebs. Due to the rapid and variable nature of blebs’ formation, their tracking is difficult and requires real-time observation [25,28]. 

The growing interest in research conducted in anchorage-independent, three-dimensional systems revealed the necessity of designing and creating markers for visualization processes conducted in such conditions. This type of study is a great alternative to traditional research based on the cell cultures conducted in monolayer. Furthermore, 3D cultures allow for a decrease in the number of in vivo studies and, therefore, seem to be the future of drug screening [29]. Despite these advantages, the studies of cellular processes under nonattachment conditions remain challenging. Membrane blebbing, cell-to-cell contact, and formation of structures such as spheroids and organoids are still difficult to visualize. In particular, real-time fluorescent visualization of membrane-related changes is a great challenge. Such research needs to use nontoxic compounds. The perfect dye is expected to be useful for real-time observation in prolonged incubation time and exhibits a stable fluorescent signal without residual background fluorescence. Small fluorophores [30,31] seem to be a great option for staining membranous cell compartments. Among others, small dyes based on benzothiadiazole [32,33], nitrobenzodiazole [34], squaraine [35], styrylpyridine [36,37,38,39], oxazolopyridine [40], perylene [41], and fluorene [42] have been proposed as membrane markers. The typical dye designed for this purpose contains a lipophilic fragment, enabling incorporation into the bilayer, and a hydrophilic part which allows for good solubility in biological systems.

Due to some limitations of colocalization experiments, different excitation and emission wavelength ranges are required for various experiments. Certain green-emitting oligoelectrolytes for membrane staining, containing a phenylenevinylene [43,44] or distyrylnaphthalene [45,46] core, blue chromone [47], red-emitting styrylpyridine [37], or dithienothiophene-based [48] dyes, have been proposed previously. In the current research, a new fluorescent dye based on a dithienothiophene S,S-dioxide core is presented as a suitable tool for live cell imaging in 2D and 3D cell culture systems.

Dithienothiophene-based (DTT) compounds have been designed and synthesized mainly as organic electronic materials. They constitute attractive objects for supramolecular chemistry [49,50,51,52]; however, some of them have also been found to be good candidates for biological applications [53,54,55,56]. Among this group of compounds, dithienothiophene (DTT) and its *S,S*-dioxide (DTTDO) derivatives have been successfully applied in cellular studies [48,57,58,59,60]. In the current manuscript, a new DTTDO derivative, named **DTTDO-NMe_3_^+^**, is proposed as an effective dye for staining cellular membranes. Its synthesis, cellular uptake, intercellular localization, and impact on cell viability are demonstrated herein. Labeling was performed in living cells under different cell culture conditions, proving the usefulness of **DTTDO-NMe_3_^+^** for membrane staining in various experimental procedures. 

## 2. Results and Discussion

### 2.1. Synthesis of Dithienothiophene S,S-Dioxide Derivatives 

**Scheme 1:**
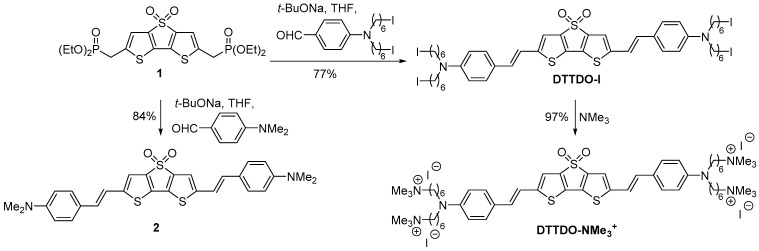
Synthetic pathway to **DTTDO-NMe_3_^+^** and DTTDO derivative **2**.

The synthesis of **DTTDO-NMe_3_^+^** and DTTDO derivative **2** was based on the bisphosphonate **1** used as a common starting material (Scheme 1). **DTTDO-NMe_3_^+^** was obtained in the reaction sequence encompassing Horner–Wadsworth–Emmons olefination reaction of bisphosphonate **1** with 4-(*N,N*-bis(6-iodohexyl)amino)benzaldehyde [48] followed by quaternization reaction of formed tetraiodide **DTTDO-I** with an excess of trimethylamine. On the other hand, compound **2**—which can be considered as a simplified derivative of **DTTDO-NMe_3_^+^** in which the four substituted alkyl chains were replaced with methyl groups—was prepared in the reaction of bisphosphonate **1** with 4-(*N,N*-dimethylamino)benzaldehyde. DTTDO derivative **2** was used only as a model compound for the comparison of its optical and electronic properties with the results obtained by theoretical calculations. 

### 2.2. Photophysical Properties of DTTDO Derivatives

**Figure 1:**
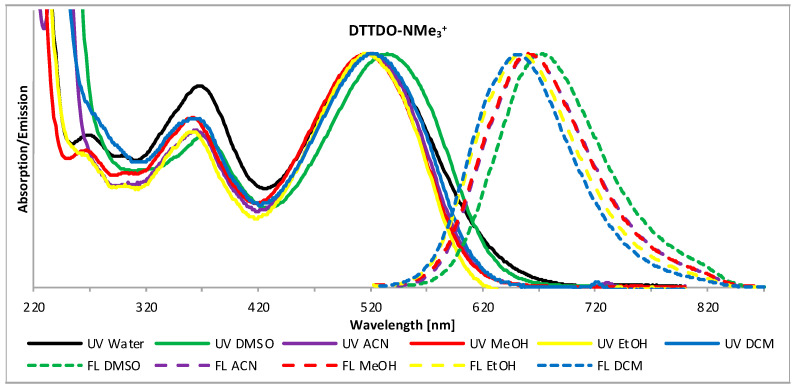
Normalized fluorescence (dotted line) and absorption (solid line) spectra of **DTTDO-NMe_3_^+^** in different solvents.

**Figure 2:**
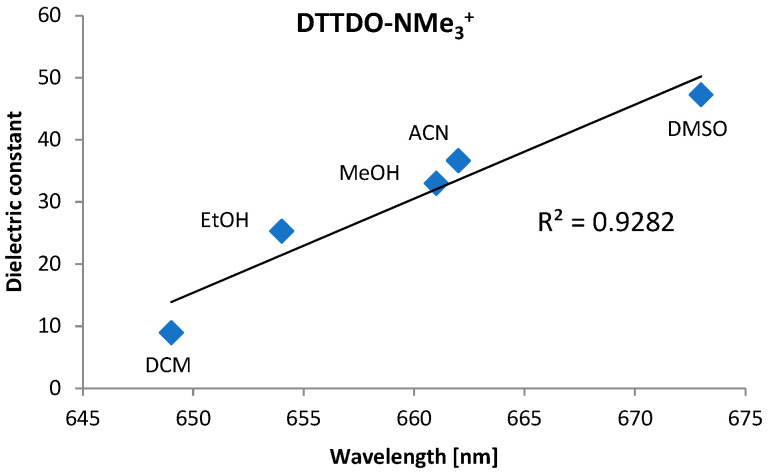
Correlation between maximum emission wavelength of **DTTDO-NMe_3_^+^** and solvent dielectric constant.

Due to the presence of four ammonium groups, **DTTDO-NMe_3_^+^** is well soluble in protic and aprotic highly polar solvents, slightly soluble in DCM, and not soluble in solvents of low polarity such as hexane, diethyl ether, or THF. Electronic absorption and emission spectra of **DTTDO-NMe_3_^+^** in different solvents are presented in Figure 1, while the maxima absorption and emission wavelengths are collected in Table 1. Absorption spectra of **DTTDO-NMe_3_^+^** in all investigated solvents exhibit 2 distinct signals: a major strong absorption signal at about 518 nm (534 nm in DMSO), and a less intensive higher energy band in the range of 350–372 nm. Emission spectroscopy revealed that **DTTDO-NMe_3_^+^** does not fluoresce in water but shows fluorescence in other solvents. In contrast to the maximum absorption wavelength, which to a large extent does not depend on the type of solvent, in fluorescence spectra of **DTTDO-NMe_3_^+^**, a strong correlation between the maximum emission wavelength and the solvent polarity was observed (Figure 2). An increase in the solvent dielectric constant resulted in a strong bathochromic shift of maximum *λ*_em_ to a longer wavelength. This observation indicates that the excited state of **DTTDO-NMe_3_^+^** is more polarized than the ground state and, consequently, it is better stabilized by polar solvents. Solvent solutions of **DTTDO-NMe_3_^+^** exhibited a significant difference between maximum λ_em_ and λ_abs_ wavelength, ranging from 132 to 145 nm, which suggests a strong electron reorganization between S_0_ and S_1_ state. The highest λ_em_ of 673 nm was observed in DMSO. The solvent polarity also strongly affects the fluorescence quantum yield of **DTTDO-NMe_3_^+^**. While in water solution, this compound does not show fluorescence, and in DMSO, the fluorescence quantum yield is only about 4%, it noticeably increases in dichloromethane, reaching a value of 17%. 

**Figure 3:**
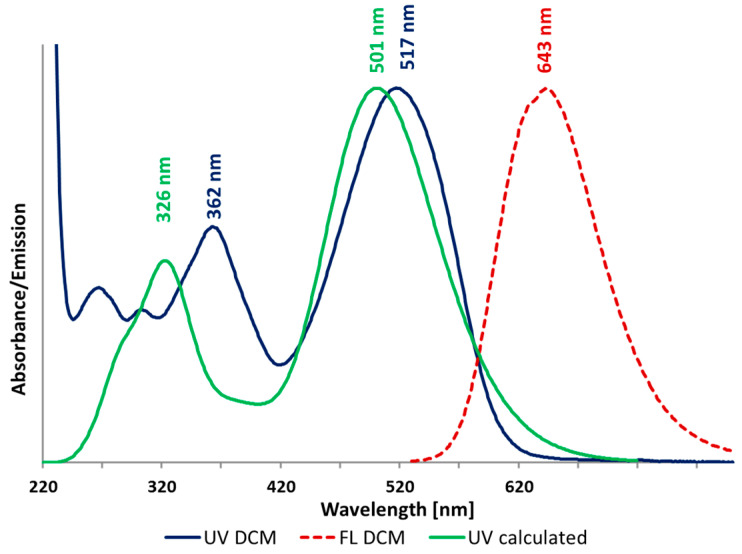
Normalized simulated absorption spectrum (green), and experimental absorption (purple) and emission (red) spectra of compound **2** in DCM.

To provide a deeper insight into the electron excitation process, time-dependent DFT calculations were conducted at the m062x/6-311+G(2d,p)//m062x-D3/6-311G(d,p) level of theory and compared with experimental results for model DTTDO derivative **2**. As evidenced by Figure 3, the calculated UV-VIS spectrum correlates well with the experimental result in terms of the shape and band intensities. Excitation bands for 2 main signals are blue-shifted by 16 and 36 nm in comparison with the corresponding experimental values. 

**Figure 4:**
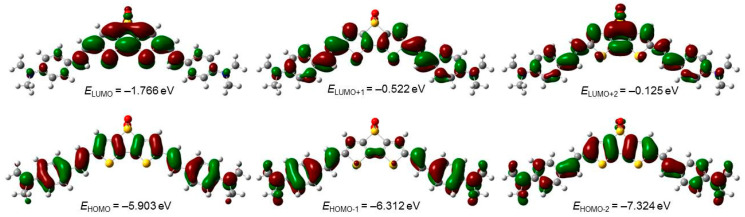
Contours (isovalue = 0.023) and energies of selected molecular orbitals of **DTTDO-NMe_3_^+^** obtained at the TDDFT m062x/6-311+G(2d,p)//m062x-D3/6-311G(d,p) level of theory in dichloromethane (SMD solvation model).

TDDFT calculation revealed that the lowest energy excitation in **2** mainly involves the HOMO–>LUMO transition (Table 2). Because the HOMO in **2** is fully delocalized in the whole molecule (except sulfur and oxygen atoms), while the LUMO displays the highest molecular orbital density on the dithienothiophene dioxide fragment, it could be deduced that this excitation has an intramolecular charge transfer (CT) character (Figure 4). A similar conclusion can be drawn from the analysis of S_0_–>S_2_ excitation, which involves the LUMO and HOMO-1 (mainly localized on the amino, styryl, and vinyl groups). On the other hand, the excitation to the S_3_ excited state has a more complex nature and encompasses the HOMO–>LUMO+2, HOMO-2–>LUMO, and HOMO-1–>LUMO+1 transitions. The analysis of TDDFT calculations also revealed that the change in electron distribution during the photoexcitation process is reflected in the change of electric dipole moment between ground and excited states. Thus, while the dipole moment for the ground state is equal to 9.68 D, its value increases by about 3 to 4 D for the first 3 excited states. These results are in full agreement with the experimentally observed bathochromic shift in the emission spectra of **DTTDO-NMe_3_^+^**.

### 2.3. Verification of Fluorescent Properties inside the Cells

**Figure 5:**
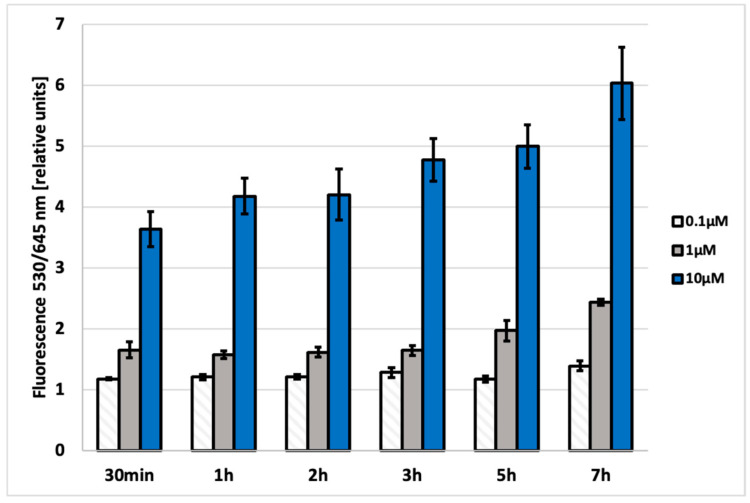
The intracellular fluorescence intensity measured at emission wavelength *λ*_em_ = 645/40 nm (*λ*_ex_ = 530/25 nm) in A431 cells incubated with **DTTDO-NMe_3_^+^** derivative for 30 min, 1 h, 2 h, 3 h, 5 h and 7 h, respectively. Concentration range: 0.1–10 µM. The bars represent the mean fluorescence value calculated with reference to the control sample (cells untreated with **DTTDO-NMe_3_^+^**).

In order to investigate the potential usefulness of **DTTDO-NMe_3_^+^** for cell staining, fluorescence measurements from the cells and microscopic observation were performed. For the quantification of intracellular fluorescence intensity, A431 cells were treated with the tested compound, washed, and fluorescence in the wavelength range characteristic for this dye (λ_ex_ = 530/25 nm and λ_em_ = 645/40 nm) was measured. To determine the conditions required for effective cell staining, the samples’ fluorescence was measured after 30 min, 1 h, 2 h, 3 h, 5 h, and 7 h upon treatment of cells with **DTTDO-NMe_3_^+^** at different concentrations (0.1, 1, and 10 μM) (Figure 5).

These time- and concentration-dependent measurements allowed for the determination of optimal conditions for cell staining. It was found that 30 min incubation with 10 μM **DTTDO-NMe_3_^+^** is sufficient to observe high fluorescence intensity derived from intracellular staining. The tests were performed in a complete culture medium, which proved that staining can be performed under standard culture conditions in the presence of fetal bovine serum (FBS) and antibiotics. 

**Figure 6:**
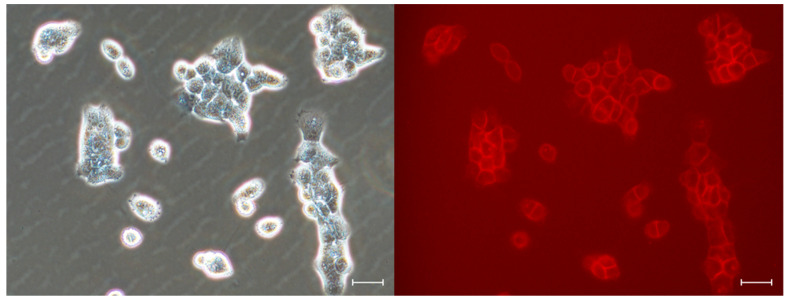
Fluorescent microscopy observation of living human A431 after 30 min incubation with 10 μM **DTTDO-NMe_3_^+^** in a complete medium containing FBS and antibiotics. Imaging was performed without washing the cells. Left panel, phase contrast; right panel, fluorescent images with TxRed filter (λ_ex_ = 540–580, λ_DM_ = 595, λ_BA_ = 600–660). Magnification, 20×; scale bars, 30 μm.

Additional evidence of the ability of **DTTDO-NMe_3_^+^** to stain cells was provided by fluorescent microscopy observations. Even after 30 min incubation with the tested compound, strong fluorescent labeling was visible (Figure 6), which indicated the possibility of using this marker for rapid cell staining in various types of assays. Because **DTTDO-NMe_3_^+^** does not exhibit fluorescence in water, washing of the cells turned out to be unnecessary for fluorescent microscopy observations, which extends the application possibilities of the tested dye and enables the real-time observation of stained cells.

### 2.4. Impact on Cell Viability

**Figure 7:**
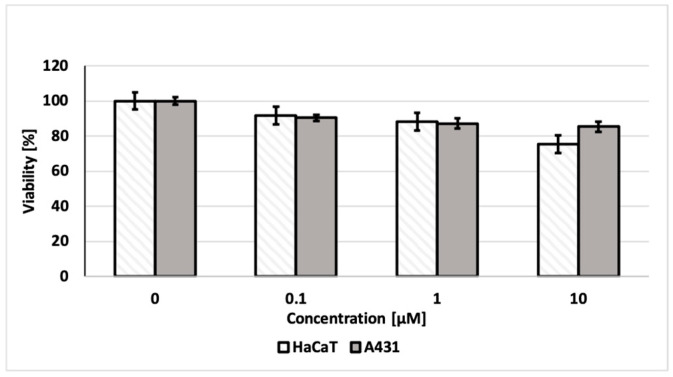
Cytotoxicity of **DTTDO-NMe_3_^+^** after 24 h treatment of A431 and HaCaT cells assessed by MTT assay. The values obtained for cells treated only with DMSO (without the addition of **DTTDO-NMe_3_^+^**) were taken as 100% viability.

In order to assess the applicability for long-term experiments, the toxicity of the new dye was investigated. Cytotoxicity tests were performed on the epidermoid squamous carcinoma cells (A431) using noncancerous cells—human keratinocytes (HaCaT). In none of these cases did cytotoxicity exceed 25% after 24 h of incubation (Figure 7). These results indicate that **DTTDO-NMe_3_^+^** can also be safely used in various types of experiments, including procedures that require long-term staining, such as real-time observations of cell properties under various conditions.

### 2.5. Intercellular Localization Studies

**Figure 8:**
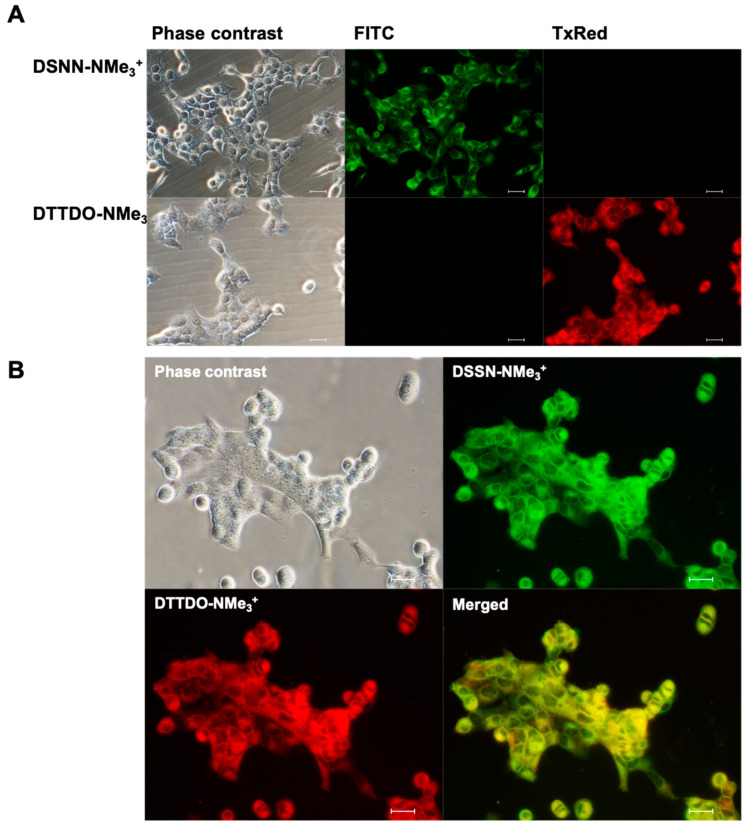
Microscopy observation of A431 cells treated with **DTTDO-NMe_3_^+^** derivatives and membranous dye—DSSN-NMe_3_^+^—at concentration of 10 μM after 24 h incubation. The DTTDO derivative was visualized with TxRed filter (λ_ex_ = 540–580, λ_DM_ = 595, λ_BA_ = 600–660), whereas DSSN-NMe_3_^+^ was visualized with FITC filter (λ_ex_ = 465–495, λ_DM_ = 505, λ_BA_ = 515–555). (**A**) The assessment of the usefulness of both dyes for colocalization studies, (**B**) colocalization experiment. The scale bars represent 30 μm.

To confirm the intercellular localization of fluorescent **DTTDO-NMe_3_^+^** dye, colocalization studies with a membrane marker—**DSSN-NMe_3_^+^** were conducted. **DSSN-NMe_3_^+^** is a conjugated oligoelectrolyte whose ability to stain the outer cellular membrane and intercellular membranous structures has been reported previously [43,44]. The large difference in the emission wavelength of these two compounds eliminated possible spectral overlapping and ensured the credibility of colocalization results. The usefulness of both dyes for colocalization studies was confirmed through their observation with appropriate filters (Figure 8A).

The co-staining studies revealed the presence of fluorescent signal in the same cellular compartments after treatment with **DTTDO-NMe_3_^+^** and **DSSN-NMe_3_^+^** membrane dye (Figure 8B). Such colocalization of the tested compound with the marker of membranous structures confirmed their localization inside the membranes, which indicated the accuracy of the research hypothesis regarding the intercalation of DTTDO derivative into lipid bilayers and showed the usefulness of the tested compound for staining cellular membranes and membranous structures. 

**Figure 9:**
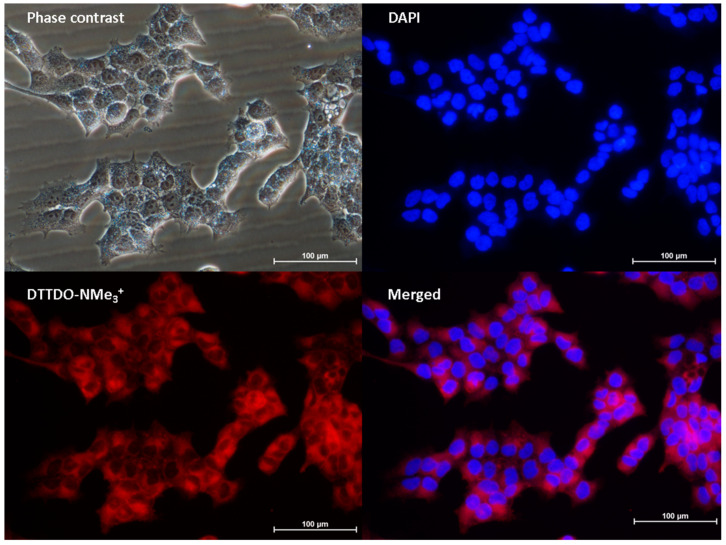
Colocalization studies of **DTTDO-NMe_3_^+^** and DAPI in A431 cells. **DTTDO-NMe_3_^+^** concentration, 10 μM; incubation time, 24 h. The DTTDO derivative was observed using TxRed filter (λ_ex_ = 540–580, λ_DM_ = 595, λ_BA_ = 600–660), whereas DAPI was observed using DAPI filter (λ_ex_ = 340–380, λ_DM_ = 400, λ_BA_ = 435–485). Scale bars, 100 μm.

Furthermore, the microscopic analysis indicated the absence of the tested DTTDO derivative inside the nucleus. In order to verify this observation, additional colocalization studies using DAPI—a cell nucleus staining marker—were performed. Obtained results confirmed that, similarly to **DSSN-NMe_3_^+^,** the tested **DTTDO-NMe_3_^+^** did not penetrate into the nucleus (Figure 9).

### 2.6. Cell Staining in 3D Cell Culture Conditions

After confirmation of the applicability of the tested DTTDO derivative for staining living cells in standard conditions, we evaluated the possibility of using this compound for dyeing cells cultured without attachment. The 3D model of cell culture is the most suitable method for in vitro studies of processes related to cancer metastasis and drug resistance. Numerous drugs which exhibited activity in traditional monolayer cell cultures (2D) were found to be inactive in further in vivo research [61,62]. Therefore, studies based on non-flat cell cultures involving spheroids, organoids, or other 3D cell culture models have become more popular recently [63,64,65,66]. Although three-dimensional cell cultures are the most suitable for studying new drugs and processes, which occur inside organisms, their applicability is still a challenge.

**Figure 10:**
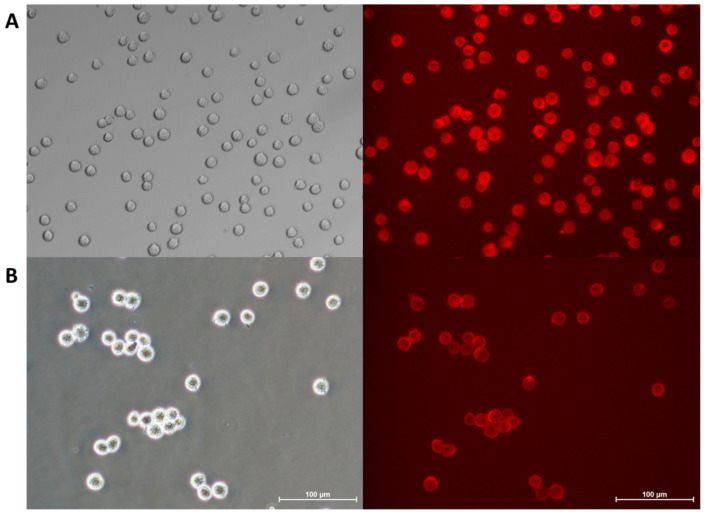
Visualization of cellular membranes in living (**A**) A431 and (**B**) MG63 cells in detached conditions. Images were captured after 30 min incubation of cells with 10 μM **DTTDO-NMe_3_^+^** derivative using TxRed filter (λ_ex_ = 540–580, λ_DM_ = 595, λ_BA_ = 600–660). Incubation was performed in full medium without a washing step. Scale bars, 100 μm.

The proposed studies showed that, regardless of the differences in the specificity of membranes in the cells, which are placed in 2D and 3D conditions, **DTTDO-NMe_3_^+^** was able to penetrate and efficiently stain membranes of both attached (Figure 6) and detached (Figure 10) cells. The lack of necessity to remove the dye from the culture medium prior to microscopic observation is a significant advantage of the tested compound, as this step is difficult to perform during real-time experiments in detached conditions. This result also indicates the potential utility of this dye for the visualization of membrane-dependent processes under detachment conditions.

**Figure 11:**
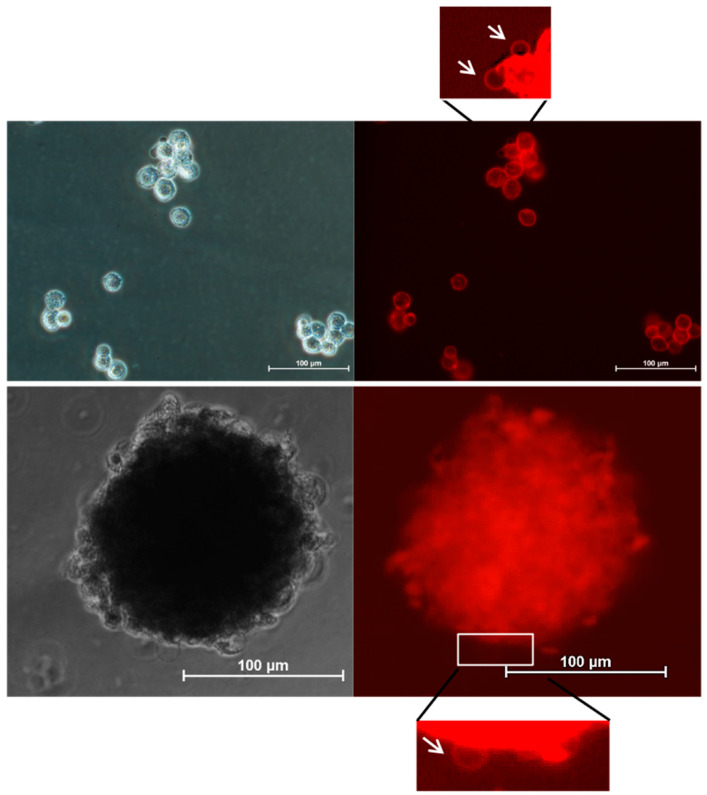
Visualization of membrane blebs (white arrows) in MG63 cells cultured in 3D conditions. Images were captured after 30 min incubation of cells with 10 μM **DTTDO-NMe_3_^+^** using TxRed filter (λ_ex_ = 540–580, λ_DM_ = 595, λ_BA_ = 600–660). Incubation was performed in full medium without a washing step. Scale bars, 100 μm.

One of the most important changes in membrane structure related to various cellular states is the formation of protrusions. Visualization of membrane blebbing may be an important tool for monitoring a wide range of processes such as apoptosis, anoikis resistance, motility, and metastasis. Similar to the green distyrylnaphthalene (DSNN) derivative [46], the tested **DTTDO-NMe_3_^+^** was found to be useful for the visualization of membrane blebs (Figure 11), which proved its potential applicability for tracking this type of membrane alteration.

Obtained results indicate that **DTTDO-NMe_3_^+^** may be used for staining cell membranes in a wide spectrum of different conditions, which makes it an effective dye for labeling membranous-dependent processes for both rapid cell visualization and long-term experiments. Moreover, this dye may be used not only for staining adherent cells in standard cell culture in monolayer but also for three-dimensional cell cultures. The ability to stain cell protrusions also makes it a good candidate to visualize any processes connected with these alterations, such as bleb-associated cell motility or blebs formation associated with anoikis, apoptotic blebbing, epithelial-mesenchymal transition-associated changes or related purposes.

## 3. Materials and Methods

### 3.1. Synthesis of DTTDO Derivatives

#### 3.1.1. Experimental Remarks

All reactions were carried out under an argon atmosphere. Commercial-grade reagents and solvents were used without further purification, except for THF, which was dried by distillation over Na/benzophenone before use. NMR spectra were recorded on Bruker AV Neo 400 (Bruker, Billerica, MA, USA) spectrometer. Chemical shifts of ^1^H and ^13^C are reported relative to the residual proton resonance in the deuterated solvents. Chemical shifts (δ) are provided in ppm. HRMS measurements were performed on a Finnigan MAT 95 (Thermo Fisher Scientific, Waltham, MA, USA) or Waters Synapt HDMS (Waters Corporation, Milford, MA, USA) mass spectrometer. Melting points are uncorrected. 4-(*N*,*N*-bis(6-iodohexyl)amino)benzaldehyde [45], 4-(*N*,*N*-dimethylamino)benzaldehyde [67], 2,6-bis[(diethoxyphosphoryl)methyl]dithieno[3,2-*b*:2′,3′-*d*]thiophene-4,4-dioxide [48] and 4,4′-bis(4′-{*N,N*-bis[6”-(trimethylammonium)hexyl]amino}styryl)stilbene (**DSSN-NMe_3_^+^**) [68] were prepared according to procedures outlined in the literature. NMR spectra of **DTTDO-NMe_3_^+^** and DTTDO derivative **2** are available in Supplementary Materials. 

#### 3.1.2. Synthetic Procedures

**2,6**-**Bis(4-{*N,N*-bis[6-(trimethylammonium)hexyl]amino}styryl)dithieno[3,2-*b*:2′,3′-*d*]thiophene-4,4-dioxide (DTTDO-NMe_3_^+^)**: A solution of **DTTDO-I** (44 mg, 0.034 mmol) in THF (10 mL) was stirred with trimethylamine (1 mL, 33 wt.% solution in ethanol) for 24 h at room temperature. The solvents were evaporated, the residue was dissolved in methanol (10 mL), and another portion of trimethylamine (1 mL, 33 wt.% solution in ethanol) was added. The reaction mixture was stirred for another 24 h. After removal of the solvents under reduced pressure, the crude material was purified by precipitation with diethyl ether from methanol solution, producing **DTTDO-NMe_3_^+^** (50 mg, 97%) as a dark purple solid. Mp 173–5 °C; ^1^H NMR (400 MHz, DMSO-*d*_6_) δ 7.47 (s, 2H, *H*C_DTTDO_), 7.39 (d, *J* = 8.4 Hz, 4H, *H*C_Ph_), 7.15 (d, *J* = 16.1 Hz, 2H, C*H*=CH), 7.00 (d, *J* = 16.0 Hz, 2H, C*H*=CH), 6.66 (d, *J* = 8.6 Hz, 4H, *H*C_Ph_), 3.28 (br s, 16H, C*H*_2_N and C*H*_2_N^+^), 3.06 (s, 36H, C*H*_3_N^+^), 1.84–1.20 (m, 32H, C*H*_2_); ^13^C NMR (101 MHz, DMSO-*d*_6_) δ 149.47 (2C, *C*_DTTDO_), 148.03 (2C, N*C*_Ph_), 142.02 (2C, *C*_DTTDO_), 131.34 (2C, *C*_DTTDO_), 130.85 (2C, *C*H=CH), 128.38 (4C, H*C*_Ph_), 122.58 (2C, CH*C*_Ph_), 115.84 (2C, H*C*_DTTDO_), 115.33 (2C, *C*H=CH), 111.50 (4C, H*C*_Ph_), 65.21 (4C, *C*H_2_N^+^), 52.17 (12C, *C*H_3_N^+^), 49.90 (4C, N*C*H_2_), 26.67 (4C, *C*H_2_), 25.91 (4C, *C*H_2_), 25.75 (4C, *C*H_2_), 22.09 (4C, *C*H_2_); HRMS (ES+) *m*/*z*: [M-I]^+^ calcd for C_60_H_98_I_3_N_6_O_2_S_3_ 1411.4067; found 1411.4047; Anal. calcd. for C_60_H_98_I_4_N_6_O_2_S_3_ (1539.28): C, 46.82; H, 6.42; S, 6.25. Found: C, 46.92; H, 6.30; S, 5.99.

**2,6-**,**Bis[4-(*N,N*-dimethyloamino)styryl]dithieno[3,2-*b*:2′,3′-*d*]thiophene-4,4-dioxide (2)**: Sodium *t*-butoxide (0.15 mL 2M solution in THF, 0.3 mmol) was added to a solution of bisphosphonate **1** (52 mg, 0.1 mmol) and 4-(*N*,*N*-dimethylamino)benzaldehyde (32 mg, 0.21 mmol) in THF (10 mL). The reaction mixture was stirred at room temperature for 4 h, neutralized with a 5% aqueous solution of HCl, and extracted with CHCl_3_ (4 × 10 mL). Combined organic layers were washed with water and dried over MgSO_4_. After solvent evaporation, the crude material was purified by column chromatography (petroleum ether/diethyl ether 3:1) to afford **2** (43 mg, 84%) as a dark red solid. Mp 217–9 °C; *R*_f_ = 0.32 (diethyl ether); ^1^H NMR (400 MHz, CDCl_3_) δ 7.36 (d, *J* = 8.3 Hz, 4H, *H*C_Ph_), 7.02 (s, 2H, *H*C_DTTDO_), 6.93 (d, *J* = 16.2 Hz, 2H, C*H*=CH), 6.87 (d, *J* = 16.0 Hz, 2H, C*H*=CH), 6.70 (d, *J* = 8.3 Hz, 4H, *H*C_Ph_), 3.01 (s, 12H, NC*H*_3_); ^13^C NMR (101 MHz, CDCl_3_) δ 150.78 (2C, N*C*_Ph_), 149.23 (2C, *C*_DTTDO_), 142.54, 132.48 (2C, *C*_DTTDO_), 131.13 (2C, *C*H=CH), 128.15 (4C, H*C*_Ph_), 124.05, 116.15 (2C, *C*H=CH and 2C, H*C*_DTTDO_), 112.40 (4C, H*C*_Ph_), 40.46 (4C, N*C*H_3_); HRMS (ES+) m/z: [M+1]^+^ calcd for C_28_H_27_N_2_O_2_S_3_ 519.1235; found 519.1235; Anal. calcd. for C_28_H_26_N_2_O_2_S_3_ (518.71): C, 64.84; H, 5.05; S, 18.54. Found: C, 64.94; H, 5.10; S, 18.42.

### 3.2. Computational Methods

Quantum-mechanical calculations were conducted using the Gaussian 09 D1 (Gaussian, Inc.: Wallingford, UK, 2009) software [69]. Geometry optimization and frequency calculation were performed with the D3 dispersion [70] corrected m062x [71] functional and Pople 6-311G(d,p) basis set [72], applying a charge density-based solvation model (SMD) [73] for dichloromethane (DCM). Vertical electronic excitation energies of the twelve lowest singlet excited states were obtained at the m062x/6-311+G(2d,p)//m062x-D3/6-311G(d,p) level of theory using the time-dependent DFT (TDDFT) method [74]. Ultrafine integration grid and tight convergence criteria for the self-consistent method were used during all calculations which were conducted without any symmetry restrictions. Atomic coordinates of DTTDO derivative **2** and partial output from TDDFT calculations are included in Supplementary Materials.

### 3.3. Optical Characterization

Electronic absorption and emission spectra were recorded on Shimadzu UV-VIS spectrophotometer UV 2700 and Horriba Scientific FluoroMax+ spectrofluorimeter, respectively. Spectrophotometric grade solvents were used in all measurements. The fluorescence quantum yield (Φ) of **DTTDO-NMe_3_^+^** was determined using Rhodamine 101 in EtOH (Φ = 1) as a reference.

### 3.4. Cell Cultures

Squamous cell carcinoma cells (A431) and human osteosarcoma cells (MG-63) were purchased from the American Type Culture Collection (ATCC, Manassas, VA, USA), while the human keratinocytes (HaCaT) were purchased from the Leibniz Institute DSMZ-German Collection of Microorganisms and Cell Cultures (Braunschweig, Germany). The A431 and HaCaT cells were cultured in Dulbecco’s Modified Eagle’s Medium (DMEM) (Biological Industries, Haemek, Israel), and human osteosarcoma (MG-63) cells were cultured in Eagle’s Minimum Essential Medium (MEM) (Sigma-Aldrich, St. Louis, MO, USA). All cell cultures were supplemented with 10% fetal bovine serum (FBS) (Sigma-Aldrich, St. Louis, MO, USA), 100 U/mL penicillin and 100 μg/mL streptomycin (Invitrogen, New York, NY, USA). Cells were cultured under standard conditions (37 °C and an atmosphere of 5% CO_2_) and passaged twice a week. Cultures were tested for mycoplasma contamination using EZ-PCR Mycoplasma Test Kit (Biological Industries, Haemek, Israel). Prior to the experiments, cells were counted using ScepterTM 2.0 Cell Counter (Merck, Darmstadt, Germany).

### 3.5. Fluorescence Measurements 

For time- and concentration-dependent intercellular fluorescence measurements, A431 cells were seeded into 96-well black plates (ThermoFisher Scientific, MA, USA) at a concentration of 10,000 cells per well and maintained under standard conditions (37 °C and 5% CO_2_) for 24 h in the cell culture incubator. Then, the tested compound was added to the cells at the following timepoints before the measurement: 30 min, 1 h, 2 h, 3 h, 5 h, and 7 h. The final concentrations of dye were: 0.1 μM, 1 μM, and 10 μM for each timepoint. Cells were incubated with tested dyes under standard cell culture conditions (37 °C and 5% CO_2_). After treatment, the medium containing **DTTDO-NMe_3_^+^** was removed and cells were washed twice with PBS buffer to prevent an eventual background signal. Then, the fluorescence intensity was measured at an excitation and emission wavelength range of *λ*_ex_ = 530/25 and *λ*_em_ = 645/40 nm using Synergy HT microplate reader (Bio-Tek, Winooski, VT, USA). The mean fluorescence of cells after treatment with the tested compound was calculated compared to the control cells (cells treated only with DMSO). 

### 3.6. Cytotoxicity

The viability of cells after treatment with the tested compound was assessed using the MTT test. The test was performed based on the previously described procedure [75]. For each experiment, cells were counted, seeded into 96-well plates at a concentration of 10,000 cells/well, and left to adhere at 37 °C and 5% CO_2_. After 24 h, the culture medium was removed and fresh medium containing **DTTDO-NMe_3_^+^** at final concentrations of 0.1, 1, and 10 µM was added. Simultaneously, negative control (cells treated only with DMSO and positive control (cells treated with SDS) were prepared. Each variant was repeated at least three times. Cells were then cultured for 24 h under standard conditions (37 °C and 5% CO_2_). After that time, the MTT reagent was added to the culture medium and cells were incubated for an additional 2 h. Then, culture medium was removed and 100 µL of isopropanol was added to each well. Plates were shaken for an hour at room temperature, and the absorbance at 570 nm and 630 nm was subsequently measured using a Synergy HT plate reader with KC4 3.2 Rev 2 software (Bio-Tek, Winooski, VT, USA). The cytotoxicity of **DTTDO-NMe_3_^+^** was estimated as a percent of living cells compared to the negative control (DMSO-treated cells) taken as 100%.

### 3.7. Detection of Fluorescence inside the Living Cell

Fluorescence microscopy observations of living cells were performed on 24-, 48- and 96-well plates. The tested compound was used at the final concentration of 10 μM. Incubation was performed for 30 min, 1 h, or 24 h, respectively. At the appropriate timepoint, the intercellular fluorescence was immediately analyzed using Nikon Eclipse microscope with TxRed filter (λ_ex_ = 540–580, λ_DM_ = 595, λ_BA_ = 600–660) without washing step. Images were captured using NisElement BR 4.30.00 software (Nikon, Tokyo, Japan).

### 3.8. Colocalization Studies

A431 cells for colocalization experiments were prepared in a similar manner to that described above. In the first stage, cells were seeded into 24-well plates at a density of 50,000 cells per well and incubated in standard conditions (37 °C, 5% CO_2_) for 24 h. After that time, medium was removed and a new medium containing only **DTTDO-NMe_3_^+^** (control sample) or **DTTDO-NMe_3_^+^** with **DSSN-NMe_3_^+^** was added to the wells. After 24 h, cells were washed with PBS buffer (Biowest, Nuaillé, France) and fixed with a 3.8% paraformaldehyde solution for 10 min at room temperature. The cells were then washed three times with PBS and intercellular fluorescence was observed using Nikon Eclipse microscope with appropriate optical filters. The distyrylstilbene derivative **DSSN-NMe_3_^+^**, which specifically stains membranous cell compartments, was visualized using FITC filter (λ_ex_ = 465–495, λ_DM_ = 505, λ_BA_ = 515–555), whereas **DTTDO-NMe_3_^+^** was observed using TxRed filter (λ_ex_ = 540–580, λ_DM_ = 595, λ_BA_ = 600–660). Data recording and image merging were performed using NisElement BR 4.30.00 software (Nikon, Tokyo, Japan).

### 3.9. Cell Staining in 3D Conditions

Three-dimensional cell cultures were conducted using Ultra-Low Attachment 96-well plates (Corning, NY, USA). Cells were washed with Dulbecco′s Phosphate Buffered Saline (DPBS) without Mg^2+^ and Ca^2+^ ions (Biowest, Nuaillé, France), detached using Trypsin-EDTA solution (Biowest, Nuaillé, France), washed, and resuspended in appropriate cell culture medium supplemented with 10% FBS (Sigma-Aldrich, St. Louis, MO, USA) and antibiotics (100 U/mL penicillin and 100 μg/mL streptomycin (Invitrogen, New York, NY, USA)). MEM (Sigma-Aldrich, St. Louis, MO, USA) or DMEM (Biological Industries, Haemek, Izrael) medium was used for MG-63 and HaCaT cells, respectively. For the preparation of spheroids, MG-63 cells were seeded into Ultra-Low Attachment 96-well plate at a concentration of 10,000 cells per well in MEM medium supplemented with 10% FBS and antibiotics. The cells were then cultured for 7 days under standard conditions (37 °C, 5% CO_2_) to form a spheroid. Then, 10 μM **DTTDO-NMe_3_^+^** was added directly to the culture medium. After 30 min of incubation, cells were placed in the standard 96-well plates (Thermo Fisher Scientific, MA, USA) and microscopy observation was performed. Both staining and microscopy observation were conducted in full medium without a washing step. Images were captured using Nikon Eclipse microscope with TxRed filter (λ_ex_ = 540–580, λ_DM_ = 595, λ_BA_ = 600–660). Data recording was performed using NisElement (Nikon, Tokyo, Japan) software.

## 4. Conclusions

In the current manuscript, the synthesis, optical properties, and biological activity of a new dithienothiophene *S,S*-dioxide derivative, named **DTTDO-NMe_3_^+^**, was presented. **DTTDO-NMe_3_^+^** was designed to penetrate and stain the cellular lipid bilayer with minimal background fluorescence. Studies of the fluorescence properties of **DTTDO-NMe_3_^+^** in different solvents confirmed its potential applicability for cell staining with different fluorescence coefficient inside the cell membranes and in the culture medium. The synthesized compound enabled efficient cell staining in whole cell medium containing serum and antibiotics under standard cell culture conditions (2D), as well as in three-dimensional cell cultures. Furthermore, due to the reduced background signal, cellular staining was possible without the need to change the medium, which allows for a variety of applications including real-time observation of membrane transformations. Colocalization studies confirmed intramembranous localization of this compound and its suitability for use in multicolor experiments with fluorescent markers which exhibit green or blue fluorescence.

Since the newly synthesized compound was planned to be used in living cells, its impact on cells’ viability was also determined. The obtained data confirmed that due to its low cytotoxicity, tested DTTDO derivative may be used for long-term incubation studies. Thus, **DTTDO-NMe_3_^+^** was found to be an effective dye which may be used for both rapid and long-term membrane staining in traditional and three-dimensional cell culture conditions.

## Figures and Tables

**Table 1:** Maximum absorption (λ_abs_), emission (λ_em_), and Stokes shift (∆λ) of **DTTDO-NMe_3_^+^** in different solvents. λ_abs_, λ_em_, ∆λ values are provided in nm.

Solvent	λ_abs_	λ_em_	∆λ
Water	519	-	-
DCM	518	650	132
ACN	519	662	143
MeOH	516	661	145
DMSO	534	673	139
EtOH	517	654	137

**Table 2:** Electronic excitation energies, oscillator strength, dipole moments, and type of major transitions for the three lowest excited states of model compound **2** obtained at the TDDFT m062x/6-311+G(2d,p)//m062x-D3/6-311G(d,p) level of theory in dichloromethane dielectric medium. ^a^ *E*_ex_—excitation energy; ^b^ λ_ex_—excitation wavelength; ^c^ μ—electric dipole moment.

Excited State	*E*_ex_ [eV] ^a^	Oscilator Strenght	λ_ex_ [nm] ^b^	μ [D] ^c^	Major Transitions (%)
S_1_	2.4766	1.6745	500.6	12.58	HOMO–>LUMO (88)
S_2_	3.2038	0.2357	387.0	14.78	HOMO-1–>LUMO (81) HOMO–>LUMO+1 (13)
S_3_	3.8045	0.7521	325.9	13.69	HOMO–>LUMO+2 (41) HOMO-2–>LUMO (30) HOMO-1–>LUMO+1 (20)

## Data Availability

Not applicable.

## Supplementary Materials

The following supporting information can be downloaded at: https://www.mdpi.com/article/10.3390/ijms24119620/s1: atomic coordinates of DTTDO derivative **2** optimized at the m062x-D3/6-311G(d,p) level of theory in DCM; partial output from TDDFT calculations; NMR spectra of **DTTDO-NMe_3_^+^** and DTTDO derivative **2**.
