# Supplementary material for: Visualization of Cellular Membranes in 2D and 3D Conditions Using a New Fluorescent Dithienothiophene S,S-Dioxide Derivative
Source: Int J Mol Sci. 2023 Jun 1;24(11):9620. doi: 10.3390/ijms24119620 (PMC10253698; doi:10.3390/ijms24119620)
Supplement: Supplementary file 1 [file ijms-24-09620-s001.zip › ijms-2399343-supplementary-final.pdf]

## Supporting Information

# Visualization of Cellular Membranes in 2D and 3D Conditions Using a New Fluorescent Dithienothiophene *S,S*-Dioxide Derivative

**Aneta Rzewnicka [1], Jerzy Krysiak [1], Róża Pawłowska [2,*] and Remigiusz Żurawiński [1,*]**

[1] Division of Organic Chemistry, Centre of Molecular and Macromolecular Studies, Polish Academy of Sciences, Sienkiewicza 112, 90-363 Lodz, Poland

2 [2] Division of Bioorganic Chemistry, Centre of Molecular and Macromolecular Studies, Polish Academy of Sciences, Sienkiewicza 112, 90-363 Lodz, Poland

## TABLE OF CONTENTS

1. Atomic coordinates of compound **2** S2
2. Partial output from TDDFT calculations S2
3. NMR spectra S5

1. **Atomic coordinates of compound 2 optimized at the m062x-D3/6-311(d,p) level of theory in dichloromethane (SMD solvation model)**

||0,1|C,0,-2.37219832,0.07350747,-2.69044705|C,0,-2.34595732,0.06656501,-1.2820429|C,0,-1.10017705,-
0.04254907,-0.72544291|S,0,0.10529631,-0.15045617,-1.94271188|C,0,-1.1015934,-0.03002768,-
3.2117402|C,0,-2.3459568,0.06656117,1.28204337|C,0,-1.10017673,-0.0425512,0.72544258|C,0,-
2.37219713,0.07350081,2.69044754|C,0,-1.10159202,-0.03003606,3.21173987|S,0,0.10529715,-
0.15046136,1.94271077|C,0,-0.75370488,-0.04097559,-4.61707822|H,0,-3.25908081,0.15535194,3.30387828
|C,0,-0.75370298,-0.04098616,4.61707776|C,0,0.49627544,-0.02593215,5.11268044|C,0,0.8771948,-
0.03724322,6.51996616|C,0,0.49627354,-0.02592748,-5.11268104|C,0,0.8771932,-0.03723864,-
6.51996669|C,0,2.22933598,0.08749449,-6.86814653|C,0,-0.042207,-0.16663138,-7.57389081
|C,0,0.35864423,-0.15924427,-8.89469796|C,0,1.7253284,-0.01720896,-9.24378767|C,0,2.6543681
,0.09944323,-8.1847072|C,0,-0.04220497,-0.16664407,7.57388965|C,0,0.35864542,-0.15925399
,8.89469706|C,0,1.7253283,-0.01720841,9.24378762|C,0,2.65436777,0.09945101,8.18470773
|C,0,2.22933638,0.08750031,6.8681468|H,0,-1.61337513,-0.04868452,-5.28090782|H,0,-1.61337304,-
0.04870115,5.28090756|H,0,1.33531802,0.01163402,4.41863712|H,0,1.33531632,0.01163264,-
4.41863761|H,0,2.96927716,0.17972105,-6.07882162|H,0,-1.09922299,-0.28208035,-7.36050972|H,0,-
0.39077595,-0.2658506,-9.66729532|N,0,2.12556565,0.00381621,-10.54862256|H,0,3.71073747
,0.19809505,-8.393411|H,0,-1.09921977,-0.28210243,7.36050786|H,0,-0.39077426,-0.26586706
,9.66729394|N,0,2.12556488,0.00381906,10.54862267|H,0,3.71073623,0.19811118,8.39341216|H,0,2.96927
714,0.17973349,6.07882226|C,0,1.15177045,-0.1639648,11.61177691|C,0,3.53588598,0.12008636
,10.86987485|C,0,3.53588741,0.12007698,-10.86987416|C,0,1.15177108,-0.16396457,-11.61177712
|H,0,1.66293645,-0.11424246,12.57045495|H,0,0.3940528,0.62618283,11.5884721|H,0,0.64053717,-
1.13082017,11.54355898|H,0,3.65363099,0.133244,11.95089137|H,0,4.11255362,-0.72200728,10.47080244
|H,0,3.96099286,1.04602052,10.46867425|H,0,3.65363292,0.13323539,-11.95089062|H,0,3.96099853
,1.0460086,-10.46867224|H,0,4.11255087,-0.72201983,-10.47080239|H,0,1.66293803,-0.11424638,-
12.57045484|H,0,0.64053327,-1.13081747,-11.54355787|H,0,0.39405701,0.62618652,-11.58847433|H,0,-
3.25908227,0.15536003,-3.3038772|S,0,-3.58501656,0.1804147,0.00000065|O,0,-4.21321423,1.49337253
,0.00000286|O,0,-4.44387803,-0.99467967,-0.0000011||Version=EM64W-G09RevD.01|State=1-A|HF=-
2536.9020247|RMSD=6.944e-009|PG=C01 [X(C28H26N2O2S3)]||@

2. **Partial output data of the TDDFT m062x/6-311+G(2d,p)//m062x-D3/6-311G(d,p) calculations in dichloromethane (SMD solvation model)**

Excitation energies and oscillator strengths:

```
 Excited State   1:      Singlet-A      2.4766 eV  500.62 nm  f=1.6745  <S**2>=0.000
    134 -> 137      -0.17875
    135 -> 138      -0.14008
    136 -> 137       0.66210
 This state for optimization and/or second-order correction.
 Total Energy, E(TD-HF/TD-KS) =  -2536.82807020
 Copying the excited state density for this state as the 1-particle RhoCI density.

 Excited State   2:      Singlet-A      3.2038 eV  386.99 nm  f=0.2357  <S**2>=0.000
    133 -> 137      -0.12118
    135 -> 137       0.63684
    136 -> 138      -0.25539

 Excited State   3:      Singlet-A      3.8045 eV  325.89 nm  f=0.7521  <S**2>=0.000
    128 -> 137       0.10237
    134 -> 137      -0.38471
    135 -> 138       0.31999
    136 -> 139       0.45137

 Excited State   4:      Singlet-A      3.8992 eV  317.98 nm  f=0.1018  <S**2>=0.000
```

134 -> 138 -0.10844
135 -> 137 0.25582
135 -> 139 0.22060
136 -> 138 0.58711

Excited State 5: Singlet-A 4.2446 eV 292.10 nm f=0.0793 <S**2>=0.000
134 -> 137 0.48579
135 -> 138 0.13603
135 -> 145 0.21116
136 -> 137 0.16567
136 -> 139 0.29004
136 -> 144 0.22112

Excited State 6: Singlet-A 4.3165 eV 287.23 nm f=0.1944 <S**2>=0.000
131 -> 137 0.10066
132 -> 138 0.10209
134 -> 145 0.12454
135 -> 144 0.41611
136 -> 138 -0.10944
136 -> 145 0.48627

Excited State 7: Singlet-A 4.3254 eV 286.64 nm f=0.1636 <S**2>=0.000
134 -> 137 -0.20915
134 -> 139 0.10324
135 -> 145 0.36396
136 -> 139 -0.23336
136 -> 144 0.43811

Excited State 8: Singlet-A 4.4437 eV 279.01 nm f=0.0089 <S**2>=0.000
134 -> 140 -0.10666
134 -> 142 -0.21700
136 -> 140 0.32450
136 -> 142 0.48212
136 -> 143 0.12887
136 -> 146 0.17595

Excited State 9: Singlet-A 4.5985 eV 269.62 nm f=0.0000 <S**2>=0.000
134 -> 143 0.11272
135 -> 141 -0.23277
136 -> 140 0.52633
136 -> 142 -0.30171

Excited State 10: Singlet-A 4.6937 eV 264.15 nm f=0.0595 <S**2>=0.000
130 -> 137 0.21447
134 -> 139 0.22890
135 -> 138 0.50811
136 -> 137 0.15448
136 -> 139 -0.22903
136 -> 149 0.14996

Excited State 11: Singlet-A 4.6983 eV 263.89 nm f=0.0298 <S**2>=0.000
134 -> 141 0.10462
135 -> 140 -0.23880
135 -> 142 0.16037
135 -> 143 -0.28926
135 -> 160 -0.11191
136 -> 141 0.51021

136 -> 159 0.12042

Excited State 12: Singlet-A 4.7865 eV 259.03 nm f=0.0005 <S**2>=0.000
134 -> 140 0.13395
135 -> 141 -0.34045
135 -> 159 -0.10499
136 -> 140 -0.17294
136 -> 143 0.49033
136 -> 160 0.12129

## 3. NMR spectra

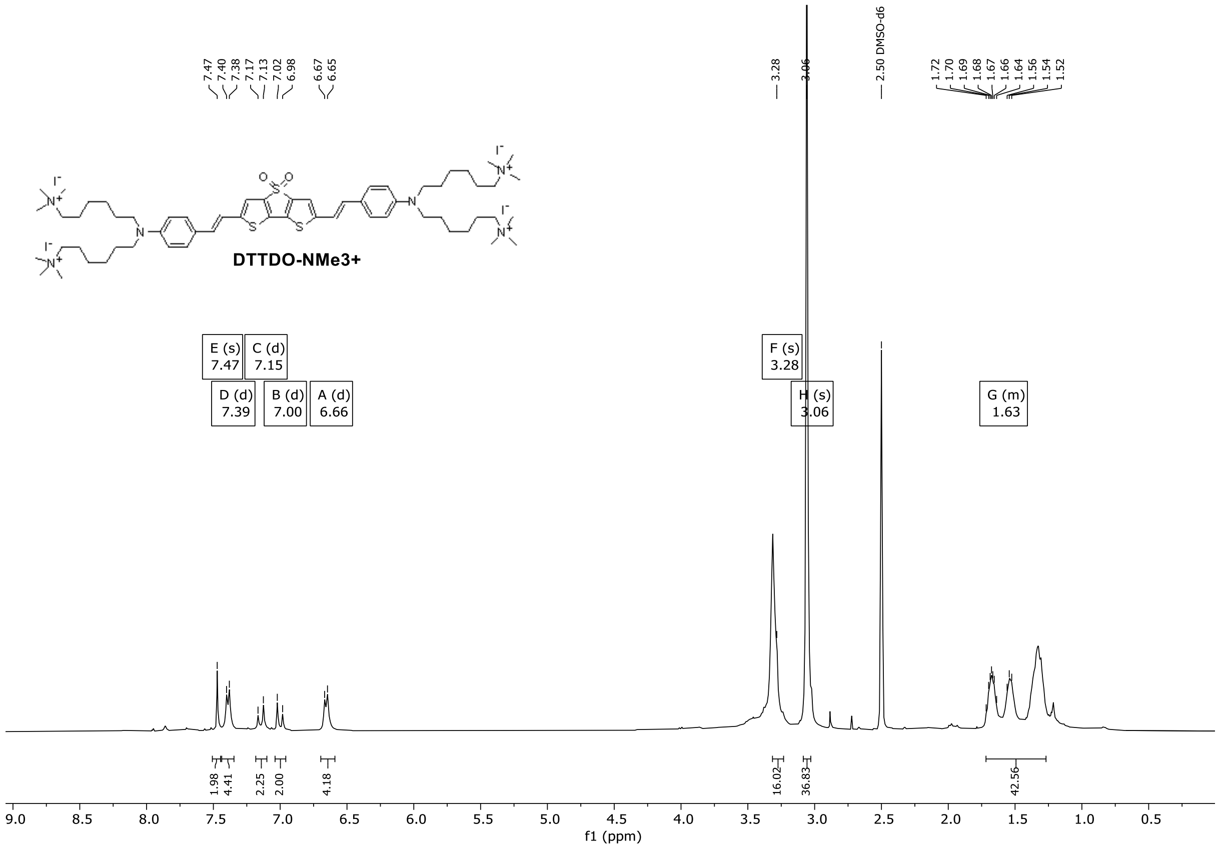


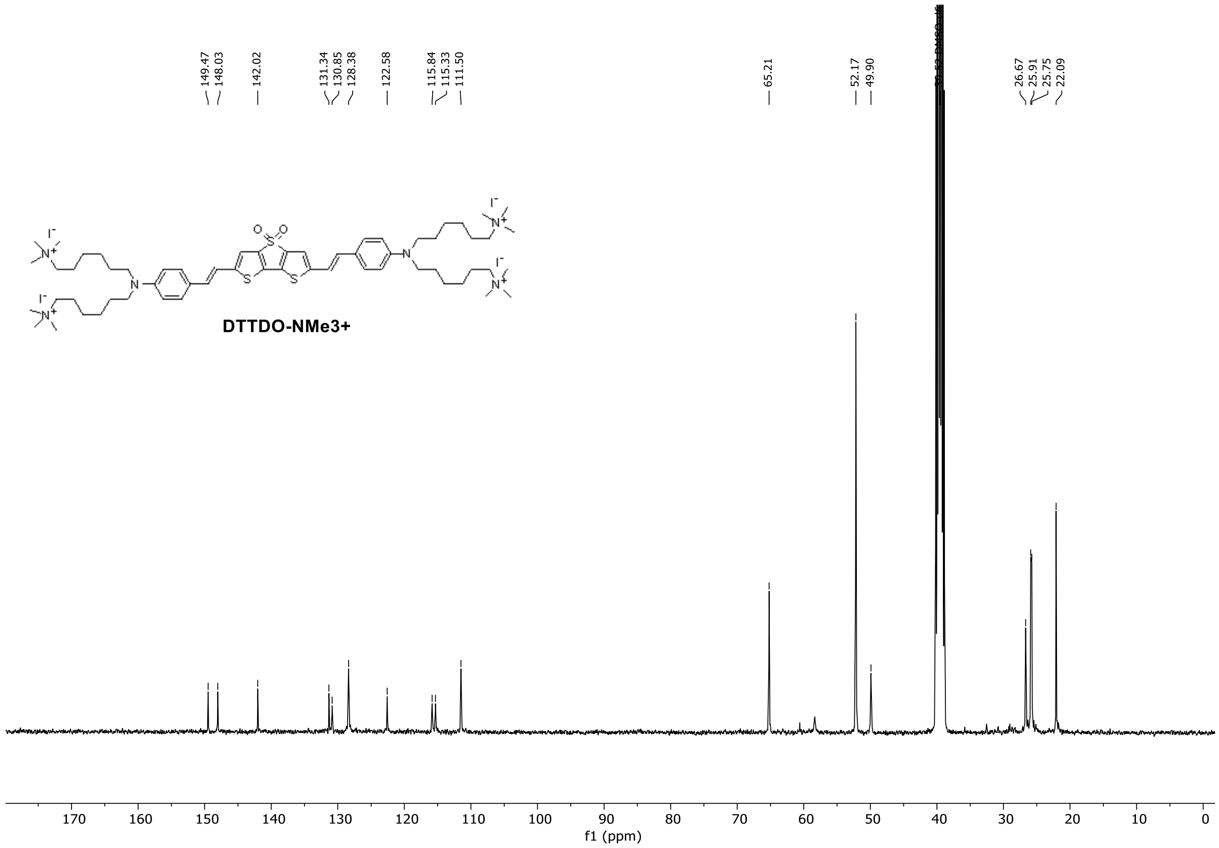

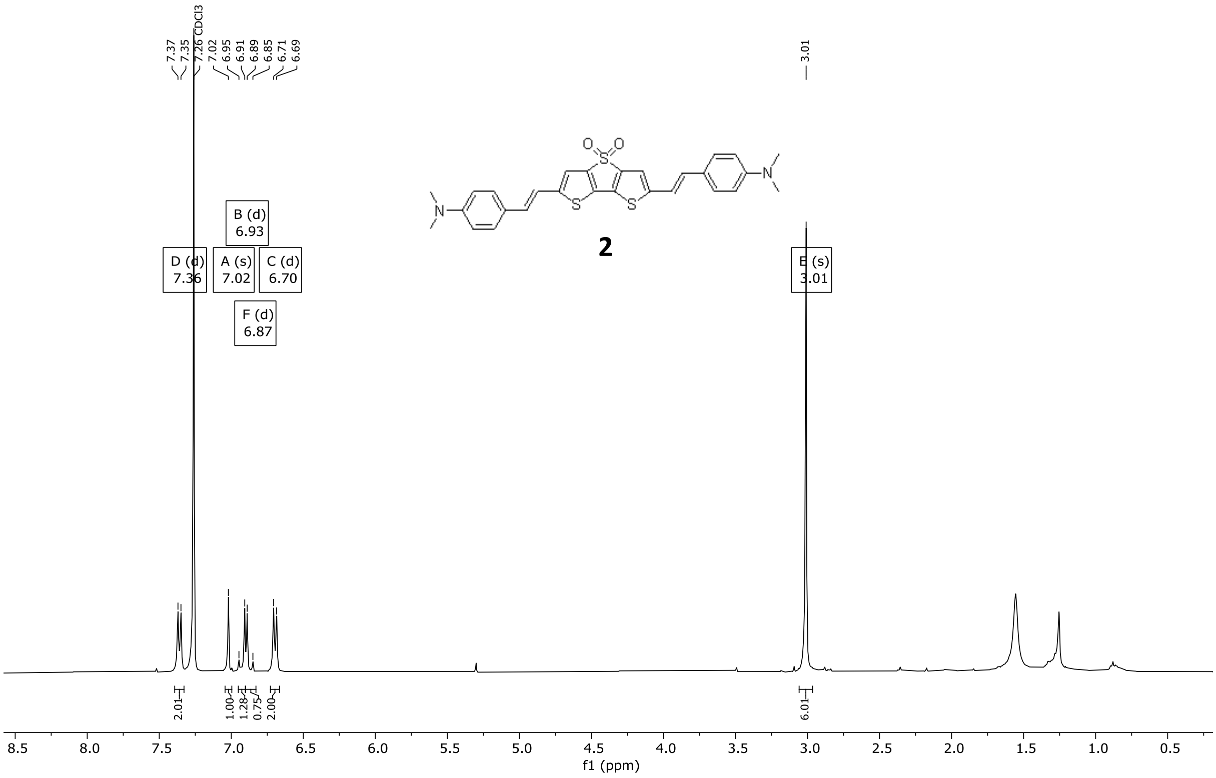
7.37
7.35
7.26 CDCl3
7.02
6.95
6.91
6.89
6.85
6.71
6.69
3.01
B (d)
6.93
D (d)
7.36
A (s)
7.02
C (d)
6.70
F (d)
6.87
E (s)
3.01
2.01
1.00
1.28
0.75
2.00
6.01
f1 (ppm)

**2**

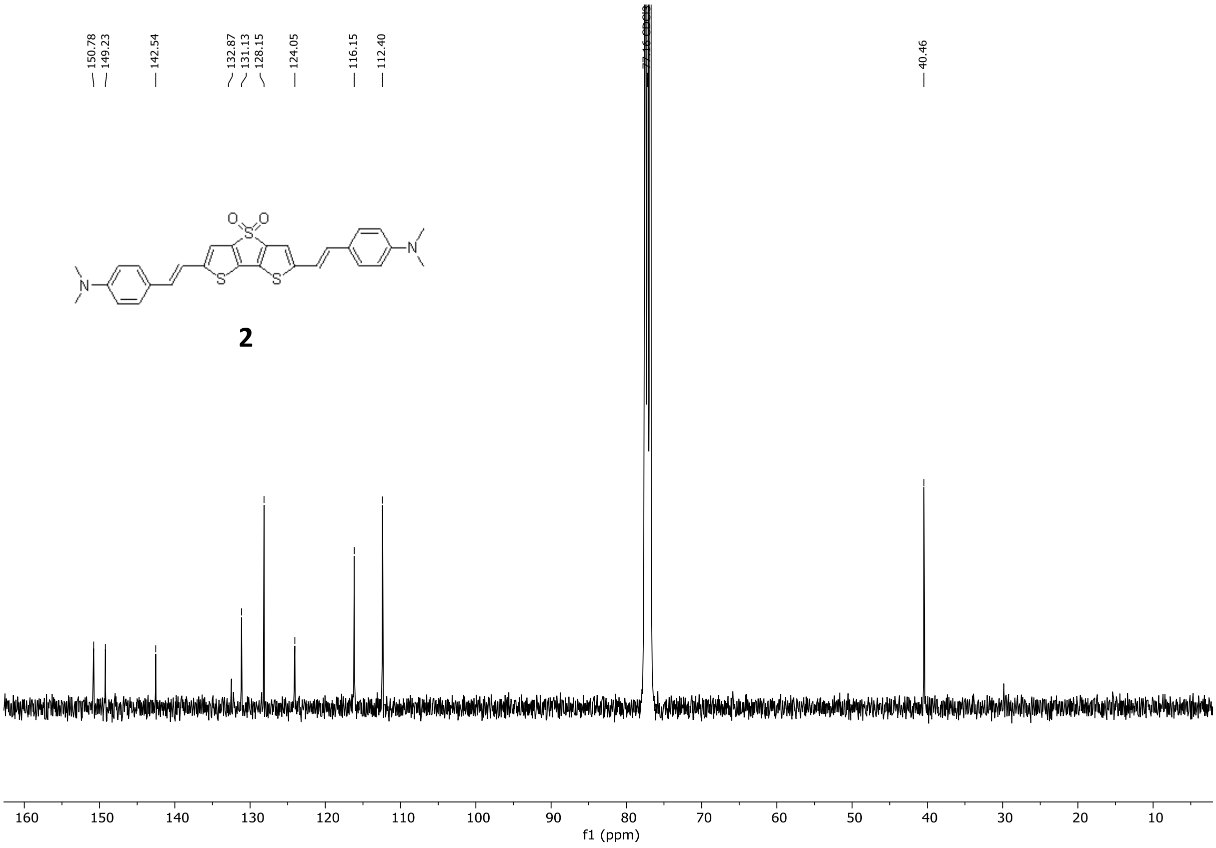
150.78
149.23
142.54
132.87
131.13
128.15
124.05
116.15
112.40
77.16 CDCl3
40.46
f1 (ppm)

**2**
